# P-1541. Process Development, Characterization, and Immunogenicity of Schistosoma haematobium Calpain-Based Vaccine Antigens

**DOI:** 10.1093/ofid/ofaf695.1722

**Published:** 2026-01-11

**Authors:** Karleen King, Adebayo J Molehin, Brooke A Hall, Deborah Oluwadare Molehin

**Affiliations:** Midwestern University, Phoenix, AZ; Midwestern University, Phoenix, AZ; Midwestern University, Phoenix, AZ; Midwestern University, Phoenix, AZ

## Abstract

**Background:**

Urogenital schistosomiasis (UGS), caused by *Schistosoma haematobium*, affects millions of people worldwide. Clinical signs of UGS include hematuria, genital lesions, infertility and bladder cancer. Experts agree that the development of an effective vaccine is integral to UGS control. Much of the schistosomiasis vaccine effort has been against *S. mansoni* with very little being done against UGS. Based on our previous work with the *S. mansoni* vaccine, Sm-p80 formulated in GLA-SE, now in clinical trials, we hypothesize that an *S. haematobium* p80 ortholog would provide protection against UGS. *S. mansoni*/animal model data indicate that resistance against schistosomiasis is partly antibody-dependent. Here, we present data on the production, characterization and immunogenicity of full-length 80 kDa *S. haematobium* calpain (Sh-p80) and 44-kDa Sh-p80-derived antigen termed, CaTaCo-Hem (Catalysis-Targeted Construct-Haematobium) formulated in TLR-4 agonist-based adjuvants, EmT4 and LiT4Q.

**Figure:**
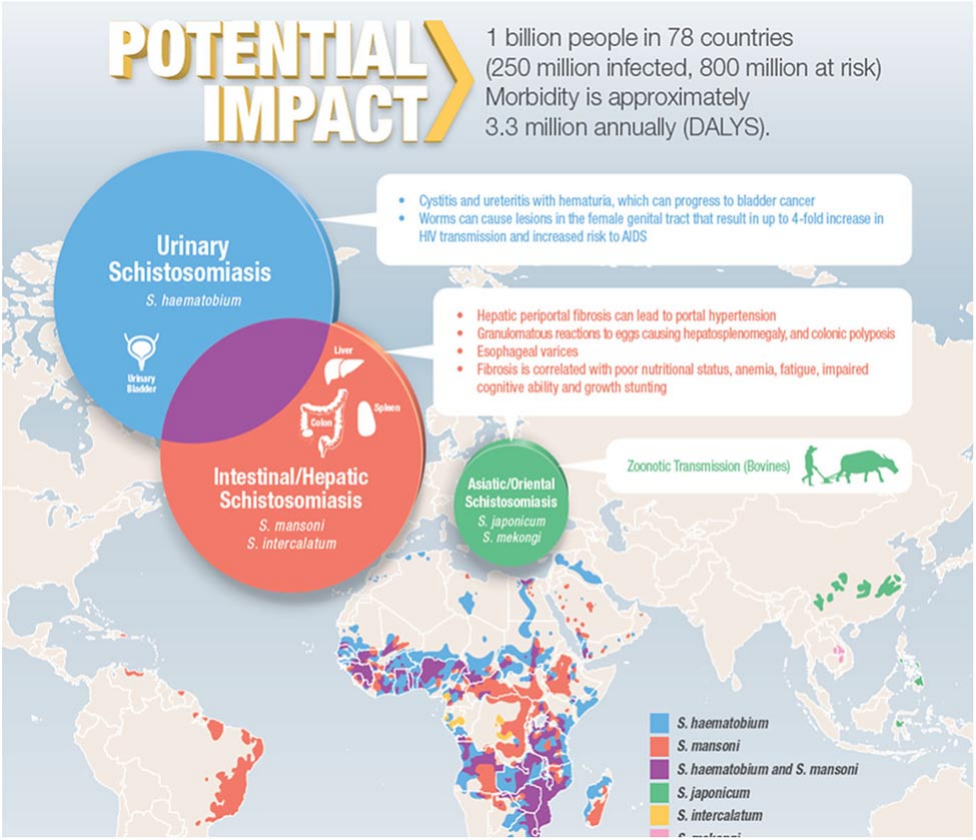
Overview of Schistosomiasis

**Figure:**
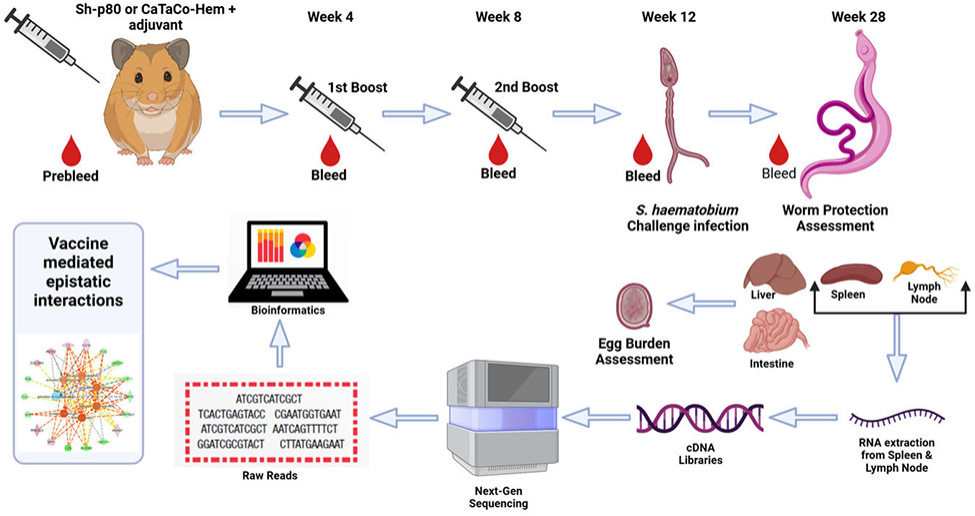
Experimental design showing immunization and blood collection schedule and necropsies

**Methods:**

Sh-p80 and CaTaCo-Hem were produced in *E. coli* and purified by affinity chromatography. Soluble deletion variants of CaTaCo-Hem were generated by deleting certain hydrophobic regions not required for enzymatic activity. Enzyme activity assessed using Calpain Glo Assay. To assess immunogenicity, 30 hamsters were divided into 6 groups (5/group) with the control groups receiving adjuvant (10 µg EmT4 or 5 µg LiT4Q) and the experimental groups receiving 100 µg Sh-p80 or CaTaCo-Hem plus either EmT4 or LiT4Q. Antibody titers were determined by ELISA.

**Figure:**
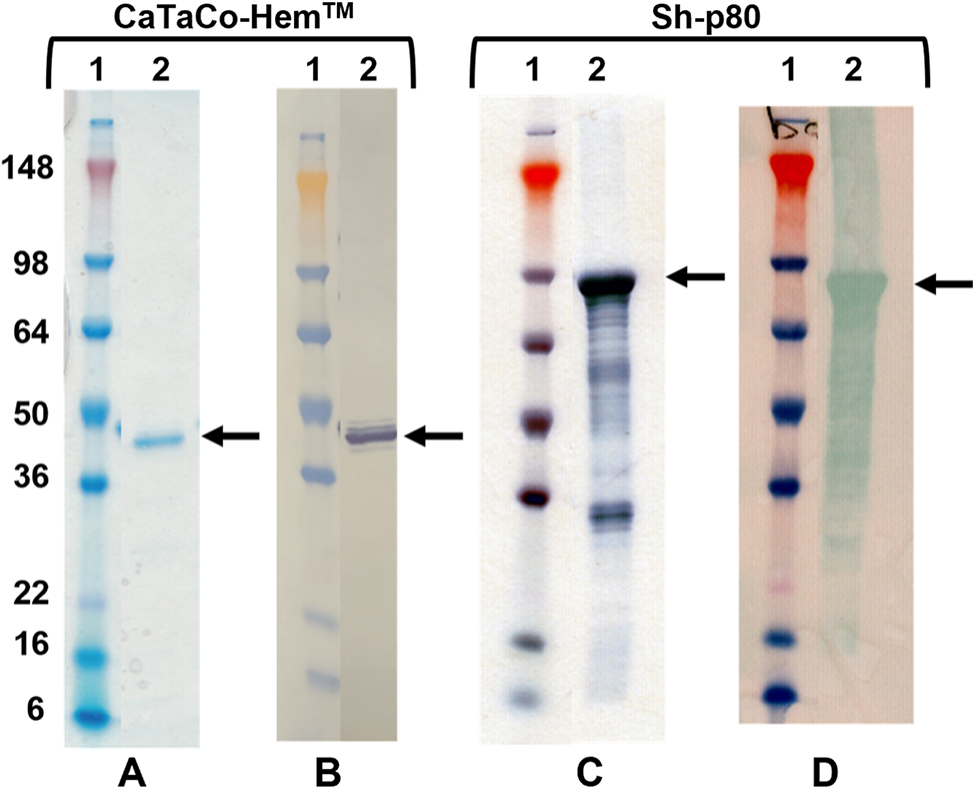
SDS-PAGE and Western Blot Analysis Analysis of recombinant proteins. (A and C) SDS-PAGE analysis of purified CaTaCo-HemTM & Sh-p80. (B and D) Western blot analysis of CaTaCo-HemTM & Sh-p80 using polyclonal anti-Sh-p80 antibody

**Figure:**
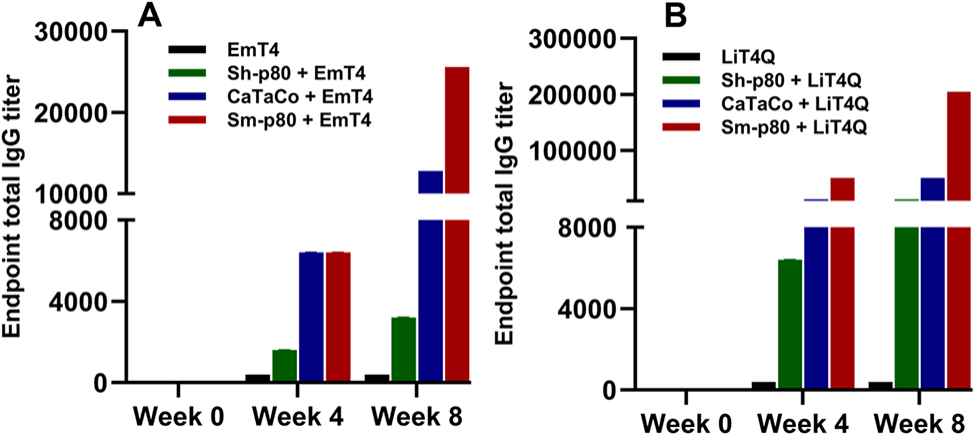
Total IgG titer results

Kinetics of antibody IgG production in immunized hamsters. (A) Total IgG titers in hamsters immunized with Sh-p80 or CaTaCo-HemTM formulated in EmT4 and (B) Total IgG titers in hamsters immunized with Sh-p80 or CaTaCo-HemTM formulated in LiT4Q. Sm-p80 (SchistoShield®) is positive control.

**Results:**

CaTaCo-Hem expressed and purified as a single monomer with no degradation/aggregation products compared to full-length Sh-p80. CaTaCo-Hem showed higher enzymatic activity than Sh-p80. ELISA data showed that CaTaCo-Hem^TM^/EmT4^TM^ and CaTaCo-Hem^TM^/LiT4Q^TM^ induced higher specific total IgG than their Sh-p80 counterparts across all timepoints measured.

**Conclusion:**

Our data showed that both Sh-p80 and CaTaCo-Hem^TM^ induced specific humoral immunity in vaccinated hamsters. The CaTaCo-Hem^TM^-based vaccine was more immunogenic than Sh-p80 and therefore could potentially be more efficacious in protection against UGS. A pilot efficacy testing of these vaccine formulations in a hamster model of infection and disease is currently ongoing.

**Disclosures:**

All Authors: No reported disclosures

